# Evaluation of relationship between endodontic access cavity types and secondary mesiobuccal canal detection

**DOI:** 10.1186/s12903-018-0570-y

**Published:** 2018-07-06

**Authors:** Gokhan Saygili, Banu Uysal, Bawar Omar, Elif Tarim Ertas, Huseyin Ertas

**Affiliations:** 1 Private Clinic, 35620 Izmir, Turkey; 2 Private Clinic, Erbil, Iraq

**Keywords:** Endodontic access cavity, Second mesiobuccally canal, Upper molar, Minimal invasive therapy

## Abstract

**Background:**

The aim of this study was to evaluate the relationship between Endodontic Access Cavity (EAC) types with MB2 canal detection ratio in the upper first molars.

**Methods:**

A total of 60 roots of extracted human maxillary first molars were selected. All teeth were prepared with Point EAC (PEAC), Conservative EAC (CEAC) and Traditional EAC (TEAC) respectively. After each group were completed, extra canal was searched. Preoperative and postoperative tooth weigh was calculated using precise scale. McNemar’s chi-square test and a paired test significant difference were used for statistical analyses.

**Results:**

The EAC types statistically were changed of tooth tissue loss quantity (*p* = 0.000). MB2 detection rate of CEAC (%53,3) and TEAC (%60) are higher than statistically that of PEAC (%31.6) (*p* < 0.05). 8 teeth MB2 canal was detected only with the CBCT images.

**Conclusions:**

In upper molars, CEAC seems reasonable in terms of detected the MB2 canal and removed hard tissue.

## Background

The fracture resistance for root canal-treated teeth is less than that of vital teeth [1]. The reason might be due to the hardness of vital dentin being more than that of endodontically dentin [2]. It was observed in previous studies that endodontic access cavity (EAC) [3] and size of its [4] are reduced to fracture resistance of tooth. Most teeth that are extracted for endodontic reasons are due to restoration causes [5]. Therefore, sound tooth tissue should be prepared as minimally invasive as possible to increase the prognosis [6].

In the upper molars, though the MB2 canal ratio depends on factors such as sex and ink [7], using various techniques and devices may affect the MB2 canal detection ratio [8]. Using an operation microscope in the root canal treatment increased the second mesiobuccal (MB2) detection ratio of the maxillary molar [9]. The MB2 may be initiated a few millimetres beneath the pulp floor [10], and specific ultrasonic devices, such as a troughing tip, were suggested for detecting such canals [11]. Minimally prepared cavities would limit the detection of the channel orifices’ localisation because molar teeth, in particular, have various canal configurations [12]. Sub-pulpal groove anatomies in maxillary first molars have indicated [13] that they have mostly trapezoid (52%) rectangle (24%) and triangle (16%) forms. The location of the canal orifices may also change if the teeth have deviation and rotation [14]. Therefore, the relationship between optimum root canal treatment and minimal invasive preparation is critical.

With the development of different devices and techniques, contemporary dentistry has seen a minimally invasive approach. Point endodontic access cavities, which are known as ninja cavities [15], are opened by removing minimum substance to reach root canals [16]. Clark and Khademi [17] have suggested a preparation technique called a conservative EAC in order to remove minimal tooth tissue. It was reported [18] that teeth with conservative EAC possess greater fracture resistance than traditional EAC. In current studies, the effect of conservative access cavity on tooth resistance have been investigated [19–21]. However, the identification of the canals especialy MB2 during conservative endodontic access cavity has not been investigated. To the best of our knowledge, there are no studies on EAC examining the different degrees of minimal preparation with the MB2 canal detection ratio. The aim of the present study was to assess the relationship between various EACs with MB2 canal detection ratios in the maxillary first molars. Second aim of this study is to investigate the weight of the tooth after changing the shape of the cavities.

## Methods

### Tooth selection

The Izmir Katip Celebi University Ethics Committee approved this study. Sixty human maxillary first molars that had been extracted for chronic apical periodontitis and that had an absence of caries and previous endodontic treatment were stored in 0.1% thymol solution at 4 °C for a maximum of six months. Calculus and tissue remnants were removed with a scaler. Then, the teeth were embedded in silicon and incubated at 38 °C for 24 h.

All images were obtained using a Cone Beam Computed Tomography (CBCT) scanner (NewTom 5G; QR, Verona, Italy). 8 × 8 cm field of view was preferred with a high-resolution denture scan mode using a 5.4-s exposure time and a 36-s scanning time. The voxel size of the images was 0.075 mm, and the slice thickness was 1.0 mm. The CBCT images of the samples were analyzed using the NNT software (New Net Technologies Ltd., Naples, FL). To interpret the radiographic images, a professional oral radiologist and an endodontist examined the images both horizontally and sagittally.

### Endodontic access cavity preparation

In this study, three different EACs were planned: Point Endodontic Access Cavity (PEAC), Conservative Endodontic Access Cavity (CEAC) and Traditional Endodontic Access Cavity (TEAC). An experienced endodontist prepared all cavities under a water spray. The MB2 canal orifice was sought with a pathfinder, and when it was found with a #10 K-file (**Mani** Inc., Tochigi Ken, Japan), the MB2 was considered detected. All teeth were prepared with PEAC, and the MB2 canals were identified. After MB2 canals were determined and recorded, the other EAC types (CEAC and TEAC) were initiated, respectively. An operation microscope (OPMI PICO; Carl Zeiss, Göttingen, Germany) was used in all cavity preparation and canal determination at magnifications ranging between × 6 and × 12.

#### PEAC

**Fig. 1:**
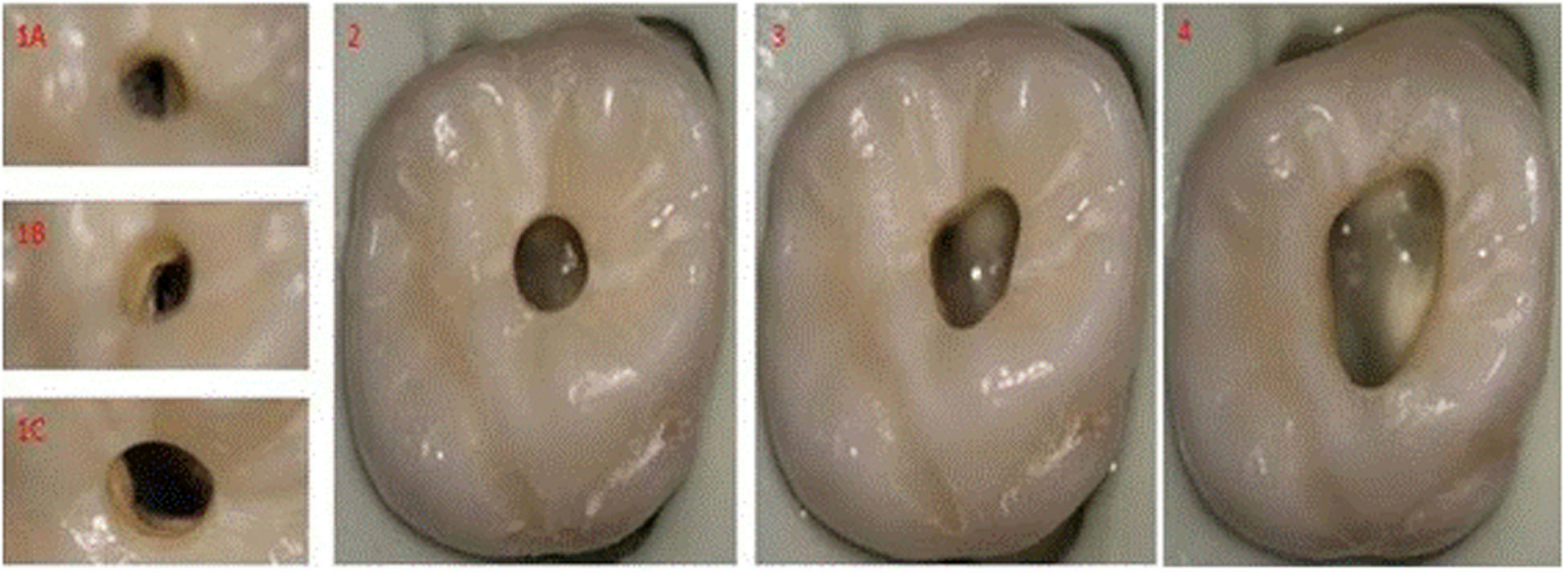
Occlusal view of cavity preperation under an operation microscope. 1A, mesiobuccal canal; 1B, distal canal; 1C, palatal canal; 2, PEAC; 3, CEAC; 4, TEAC The cavity widht of each tooth varies due to anatomic causes

The cavity was opened by using a #1014diamond round bur (#FG 1014 G, Sorensen, SP, Brazil) perpendicularly at the deepest point of the occlusal surface. After reaching the dentin, we reached the pulp using a #4 steel round burs (#CA 4 and CACH 4 Sorensen, SP, Brazil). Then, when the pulp chamber was reached, the cavity was slightly expanded buccolingually using a fissure bur. The mesiodistal length of the cavity was set to 2 mm; meanwhile, the buccolingual length of the cavity was to 3 mm. Again, the steel round bur was adjusted as 45–50 degree angle to the axial walls. The #4 steel round bur was used obliquely to straighten the inner walls. Mesiobuccal-distal and palatinal-channels were opened in a way that a #10 K-file could easily penetrate. Ultrasonic preparations were used only if the MB2 canal location could not be found. The troughing was began with a #2 Munce Discovery Bur (CJM Engineering, Santa Barbara, CA). The process started along the groove line near the MB with a trough 2–4 mm horizontally and 3 mm vertically. The MB2 orifice was searched with a Schilder plugger (Dentsply, Maillefer, Johnson City, TN, USA) and detected with a #10 K-file. An example of the PEAC is shown in Fig. 1.

#### CEAC

**Fig. 2:**
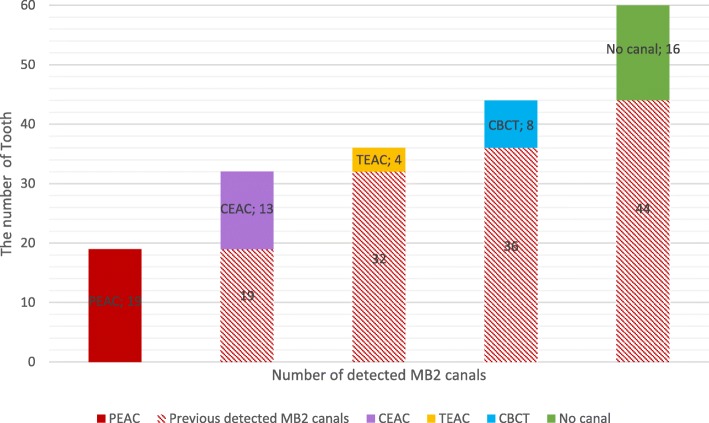
The number of detected MB2 canals with all EAC’s and CBCT images

The teeth with PEAC were then converted to CEAC. The endodontic access cavity was expanded according to the orifices of MB-P and D canals in a sequence using a #1092 fissure bur (#FG 1092G, Sorensen, SP, Brazil) and #3101 thin fissure bur (#FG 3101G, Sorensen, SP, Brazil), and a triangular form was created as shown in Fig. 2. The thin fissure bur was adjusted as 20 degree to axial wall and an inverted conic access cavity was provided. It was advanced with the angled fissure bur until the axial wall of the pulp chamber and each canal used that procedure. Irregular areas were removed using long steel burs and the cavity was completed with non–end-cutting batt bur. Finally, a mild quadrangular form was created to access the cavity by passing a fissure bur from the MB towards the mesiopalatal direction.

#### Teac

The cavity’s external outline is designed according to the base (trapezoid-rectangle-triangle) and the width of the pulp chamber. The base is towards the buccal, and the apex is to the lingual, with the canal orifice positioned at each angle of the triangle. The cavity is entirely within the tooth’s mesial half and does not invade the transverse ridge; however, it is extensive enough, buccal to lingual, to allow positioning of instruments and filling materials. Both the buccal and lingual walls slope buccally. The mesial and distal walls funnel slightly outward.

### Assessment of weight loss

Before and after the procedure, each tooth was prepared and scanned to calculate the amount of hard tissue removed. The weights of the teeth were calculated with precision scales (Precısa LS 320 A, Switzerland). Before the teeth were weighed, they were incubated at 38 °C for 24 h.

### Statistical analysis

Due to teeth being included in all three groups, each group was evaluated as 60 teeth. MB2 canals that were detected in the previous groups were accepted as diagnosed in the next group. All statistical analyses were performed with SPSS 20.0 for Windows (SPSS, Chicago, IL). The relationship between the EAC groups and the detected MB2 canals was analysed using McNemar’s chi-square test (*p* < 0.05). The weight loss for all groups was compared with a paired test.

## Results

In this study, 60 teeth were successfully prepared with PEAC, CEAC and TEAC. Though an operation microscope, ultrasonic preparation and TEAC were used, MB2 canals were not found in the eight teeth diagnosed with CBCT; the MB2 canals of these teeth were mostly seen in the middle third of the root. Figure 2 shows the distribution of detected MB2 canals with EAC and CBCT. In our study, 73.3% of the upper first molars indicated at least two MB canals.

Furthermore, there was no significant difference between CEAC and TEAC in terms of determining MB2 canals (*p* > 0.05). However, our findings have shown that the MB2 detection rate of CEAC and TEAC was statistically higher than that of PEAC (*p* < 0.05). Both EACs’ dental tissue losses are presented in Table 1. According to these findings, the EAC types were statistically changed in terms of hard tissue loss quantity (*p* = 0.000).

**Table 1:** Mean differences of dental tissue loss in milligrams, standard deviations and paired samples test *p*-values

Paired groups		Paired Differences		
		Mean	Std. Deviation	Sig. (2-tailed)
Pair 1	intact – PEAC	87	34.5	0
Pair 2	intact - CEAC	117	31.3	0
Pair 3	intact – TEAC	164	43.8	0
Pair 4	PEAC-CEAC	30	5.5	0
Pair 5	PEAC-TEAC	77	10.8	0
Pair 6	CEAC-TEAC	47	12.5	0

## Discussion

In endodontically treated teeth, the reason for long-term failure may be due to cracks [5], reduction in dentine hardness [2] and EAC width [4] effects. Improvements in magnifying visualisations and tool development means less hard tissue may be lost [22]. In the study, all teeth were prepared under an operation microscope, and it was observed that the cavity preparation was more controlled with the operation microscope.

Many researchers have investigated endodontic access cavities [15, 19–21]. Some studies reported that there is no difference between conservative endodontic access cavity and traditional cavity in term of fracture resistance [19, 21] but still the reverse of this investigation can be seen in literatüre [20]. It isn’t exactly clear that decreasing the size of endodontic access cavity reduces fracture resistance. Thus, there is still need to work on this subject. Because, the cavity walls in each cavity type are not the same when viewing photos and 3D CBCT images in all studies including this study. While we were preparing the PEAC in minimum possible way to reach the canals, we expanded CEAC cavity according to the pulp chamber and the positions of the canals..,In this study, it was seen that the TEAC group removed more hard tissue than the CEAC group. However, we prepared the smallest endodontic access cavity by means of a #10 K-file that could easily reach the apex, and we observed difference between PEAC and CEAC in terms of removed hard tissue. The amount of hard tissue removed is important because previous studies [23, 24] have shown that the fracture resistance for endodontically treated teeth significantly decreases if even a millimetre of remaining dentin is removed.

Extensive data exists regarding the MB root of the upper first molar and its number of canals [7–10]. Stropko [25] indicated that the rate of MB2 canal diagnosis can be increased by developing micro-endodontics and by using more specific tools. Although CBCT introduces an amount of radiation [26], it is one of the most reliable methods for MB2 determination [27]. The study, which used all detection possibilities (operation microscope, ultrasonic preparation, preflaring), determined that more MB2 canals were found with CBCT than that of all other groups. Verma and Love [10] observed that branching occurred in 30% of the middle and apical areas of the MB canal. Furthermore, to desirably classify the EAC, we used non-carious teeth without crown destruction. Because these teeth were extracted with the indication of chronic periodontitis, they were likely to be derived from elderly patients. Previous studies [28, 29] have reported that elderly patients have higher secondary dentin formation than young patients and that the MB2 canal may not be sufficiently identified.

In endodontics, large cavites can be opened in molar teeth due to caries, occasionally EAC can be opened on the occlusal surface of sound molar teeth due to the reasons such as periodontal, prosthetic, surgery and hypersensitivity [30]. We tried to classify three different types of EAC cavities. Contrary to PEAC, the preparations of CEAC and TEAC are a major factor in the anatomy of the pulp chamber and canals. The beginning of secondary dentin in the pulp chamber over time, the approach of the orifices of the canals and the inclination of the roots can affect the dimensions of these two types of cavities. Therefore, it is quite difficult to give a definite boundary to the occlusal region of these two types of cavities. Hence, the main criterion for determining the groups in this study is the angle between the orifices of the canals and the axial wall.

EAC may vary from patient to patient and according to tooth features such as canal length, calcification size, root curvature, etc. [31]. Recently, researchers and clinicians have designed various EAC types with minimally invasive therapy. For example, Peters and Koka [31] suggested that EAC should be advanced towards canal orifices as well as pulp chambers to prevent unnecessary loss of hard tissue. In the current study, the molar teeth were first prepared with PEAC and 31.6% of the molar teeth were diagnosed with MB2. Although there is no enough information in literature about this type of cavity, nowadays some dentists prefer to use PEAC technique to remove minimal amount of hard tissue, especially with the development of vision techniques. Round or slightly oval cavity shapes can be seen, as if indicating a minimally invasive approach. This study observed that the MB2 rate of PEAC diagnosis significantly reduced. There is a large risk of complications like fracture of files and channel transportation in teeth with PEAC due to the difficulty in finding MB2 and lack of straight line access for the file to enter the canals. In CEAC technique, the canal morphology is highly significant. However, the use of thin cylindrical burs, ultrasonic and magnification is essential for the preparation of this type of cavity. Clark [32] suggested that a root canal structure is not considered a prefabricated post, and it must be approached as a biomimetic dental structure. We can state that EAC must be arranged appropriately with the root canal anatomy to clearly see the canal orifices.

## Conclusion

In conclusion, the amount of material removed from the teeth is significantly increased when the cavity is expanded. The upper first molars should be prepared according to the tooth anatomy to determine the MB2 canals. It may not be required for the tooth be prepared with TEAC to detect the MB2 canals. However, more detailed studies are needed on the endodontic access cavity and the effects of such factors as transportation and obturation.

## Abbreviations

°C: Celsius
3D: 3 Dimensional
CBCT: Cone Beam Computed Tomography
CEAC: Conservative Endodontic Access Cavity
D: Distal
EAC: Endodontic Access Cavity
MB: MesioBuccally
MB2: Second Mesiobucally
mm: millimeter
NNT: New Net Technologies
P: Palatal
PEAC: Point Endodontic Access Cavity
TEAC: Traditinoal Endodontic Access Cavity
